# Immune modulation attenuates infantile neuronal ceroid lipofuscinosis in mice before and after disease onset

**DOI:** 10.1093/braincomms/fcab047

**Published:** 2021-03-21

**Authors:** Janos Groh, Kristina Berve, Rudolf Martini

**Affiliations:** Section of Developmental Neurobiology, Department of Neurology, University Hospital Würzburg, 97080 Würzburg, Germany

**Keywords:** infantile neuronal ceroid lipofuscinosis, neuroinflammation, immunomodulation, T-lymphocytes, neurodegeneration, attenuation of disease, preventive treatment

## Abstract

Targeting neuroinflammation in models for infantile and juvenile forms of neuronal ceroid lipofuscinosis (NCL, CLN disease) with the clinically established immunomodulators fingolimod and teriflunomide significantly attenuates the neurodegenerative phenotype when applied preventively, i.e. before the development of substantial neural damage and clinical symptoms. Here, we show that in a mouse model for the early onset and rapidly progressing CLN1 form, more complex clinical phenotypes like disturbed motor coordination and impaired visual acuity are also ameliorated by immunomodulation. Moreover, we show that the disease outcome can be attenuated even when fingolimod and teriflunomide treatment starts after disease onset, i.e. when neurodegeneration is ongoing and clinical symptoms are detectable. In detail, treatment with either drug led to a reduction in T-cell numbers and microgliosis in the CNS, although not to the same extent as upon preventive treatment. Pharmacological immunomodulation was accompanied by a reduction of axonal damage, neuron loss and astrogliosis in the retinotectal system and by reduced brain atrophy. Accordingly, the frequency of myoclonic jerks and disturbed motor coordination were attenuated. Overall, disease alleviation was remarkably substantial upon therapeutic treatment with both drugs, although less robust than upon preventive treatment. To test the relevance of putative immune-independent mechanisms of action in this model, we treated CLN1 mice lacking mature T- and B-lymphocytes. Immunodeficient CLN1 mice showed, as previously reported, an improved neurological phenotype in comparison with genuine CLN1 mice which could not be further alleviated by either of the drugs, reflecting a predominantly immune-related therapeutic mechanism of action. The present study supports and strengthens our previous view that repurposing clinically approved immunomodulators may alleviate the course of CLN1 disease in human patients, even though diagnosis usually occurs when symptoms have already emerged.

## Teaser

In models of neuronal ceroid lipofuscinoses, preventively targeting neuroinflammation attenuates neurodegeneration. Here Groh et al. show that in a model for infantile neuronal ceroid lipofuscinosis also complex clinical phenotypes like visual acuity, can be ameliorated by immunomodulation. Importantly, treatment after disease onset also mitigates disease, albeit less robustly than preventive treatment.

## Graphical Abstract

**Graphical Abstract:**
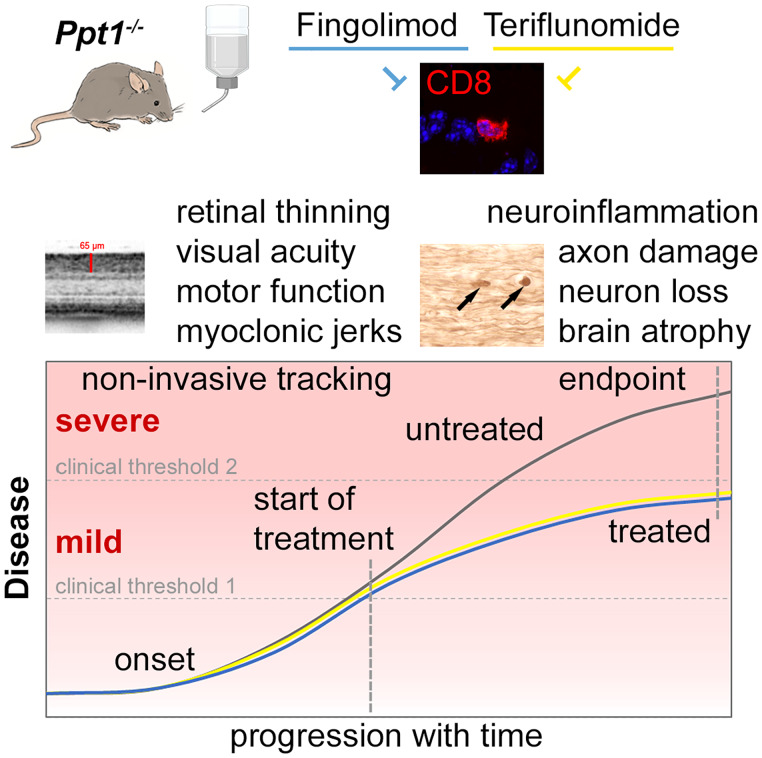

## Introduction

The neuronal ceroid lipofuscinoses (NCLs, CLN diseases) are genetically caused lysosomal storage disorders with detrimental impact on the nervous system, resulting in substantially reduced life quality and longevity.^1^^,^^2^ One of the most aggressive and rapidly progressing forms is designated infantile NCL^3^ or CLN1^2^ and caused by mutations in the *PPT1* gene, encoding for the soluble, lysosomal enzyme palmitoyl-protein thioesterase.^4^ Starting at early childhood and being a typical orphan disease lacking any causal therapy, diagnosis of this disease often drives the respective families into a hopeless and desperate situation.

Mice deficient in the respective culprit gene, *Ppt1*, also called CLN1 mice, are valuable tools to study pathogenesis and eventually to develop urgently needed treatment approaches,^5^ an unmet goal since many years. By performing genetic proof-of-principle experiments using distinct immune-mutants crossbred to *Ppt1*-deficient mice, our group has previously shown that neuroinflammation is a robust, secondary amplifier of CLN1 disease thus possibly representing a treatable target.^6^^,^^7^ Additionally, in the same model, another group showed mild mitigation of neurological features when applying the cytokine-regulatory small molecule MW151.^8^ As a potential base for an immunomodulatory approach in humans, we recently performed two therapeutic approaches in CLN1 mice, using clinically approved immunomodulators originally designed for the treatment of multiple sclerosis.^9^ In one approach, we applied fingolimod (or FTY720), a sphingosine-1-phosphate (S1P) receptor modulator, impairing lymphocyte emigration from secondary lymphatic organs and infiltration into the CNS.^10^ In the second approach, we used teriflunomide, a selective dihydroorotate dehydrogenase inhibitor attenuating proliferation and modulating mitochondrial respiration of activated, high-affinity lymphocytes.^10^^,^^11^ Although both drugs are mechanistically clearly distinct regarding their immunomodulatory functions, we found for both approaches not only reduced CNS inflammation but also a clear amelioration of histopathological changes and improved clinical outcome when the drugs have been applied before disease onset.^9^ Here, we reproduce these preventive approaches and additionally demonstrate that either drug ameliorates complex clinical phenotypes, like disturbed motor coordination and impaired visual acuity. Most importantly, we further show that the therapeutic application of the drugs, i.e. treatment of ongoing neurodegenerative disease, is a promising therapeutic option in the CLN1 model and possibly also in patients. These data are of high translational impact, since they may identify the immunomodulators as beneficial off-label medications for individual CLN1 cases as long as causal therapies are not available. Moreover, our data may be the base for urgently needed clinical trials, aiming to repurpose the clinically approved immunomodulators fingolimod and teriflunomide for mitigating devastating CLN1 disease.

## Materials and methods

### Animals

All mice were on a uniform C57BL/6J genetic background and were kept at the animal facility of the Centre for Experimental Molecular Medicine, University of Würzburg, under barrier conditions and at a constant cycle of 12 h in the light (<300 lux) and 12 h in the dark. Animal experiments were approved by the Government of Lower Franconia, Germany. *Ppt1*-deficient (*Ppt1*^−^^*/*^^−^) mice with disruption of exon 9^5^ were used as a CLN1 disease model and age-matched *Ppt1^+/+^* littermates were used as wild-type controls. Mice of either sex were analysed, since we previously did not detect prominent sex-related differences in untreated *Ppt1*^−^^*/*^^−^ mice using the here described readout parameters.^12^ Genotypes were determined by conventional PCR using isolated DNA from ear punch biopsies following previously published protocols.^5^ In some experiments in which we aimed to genetically inactivate adaptive immune reactions in the CLN1 models, *Ppt1-*deficient (*Ppt1*^−^^*/*^^−^) mice were crossbred with *Rag1*-deficient (*Rag1*^−^^*/*^^−^) mice^13^ according to previously published protocols.^6^^,^^14^*Rag1*-deficient mice lacking mature T- and B-lymphocytes do not display any neurological abnormalities and longevity, behaviour and body weights are normal under standardized conditions (specific pathogen-free).

### Immunomodulatory treatment

Fingolimod (FTY720; Sigma-Aldrich; SML0700) was dissolved in autoclaved drinking water at 3 µg per ml and provided *ad libitum*. With an approximate consumption of 5 ml per day and 30 g body weight, this corresponds to a dose of 0.5 mg/kg body weight per day. Teriflunomide (provided by Sanofi Genzyme or purchased from Biorbyt; orb146201) was dissolved in autoclaved drinking water containing 0.6% Tween 80 at 60 µg per ml, corresponding to a dose of 10 mg/kg body weight per day. These concentrations are based on previous animal experiments in our^9^ and other laboratories^15^^,^^16^ and nearly correspond to doses used for human multiple sclerosis patients, when a dose conversion scaling is applied.^17^ Non-treated controls received autoclaved drinking water without the compounds and the water with or without the compounds was changed weekly. CLN1 mice were treated preventively by starting at 1 month of age and therapeutically by starting at 5 months of age and monitored daily regarding defined burden criteria and phenotypic abnormalities. No obvious side effects were detected with both treatment approaches. Quantification of myoclonic jerks - visible as upper body contractions - in treated and untreated *Ppt1*^-/-^ mice was performed as previously described.^5^^,^^6^,^6^ At the end of the treatment, mice were euthanized with CO_2_ (according to guidelines by the State Office of Health and Social Affairs Berlin), blood was removed by transcardial perfusion with phosphate-buffered saline (PBS) containing heparin, and tissue was fixed by transcardial perfusion with 2% paraformaldehyde (PFA) in PBS. Tissue was harvested, post-fixed, dehydrated and processed as described.^6^ Before embedding of the brains, olfactory bulbs and medullae were separated at defined positions and total brains or cerebella including pontes were weighed using an analytical balance (ABT 220-5DM; Kern).

### Analysis of visual acuity

Mice were analysed regarding visual acuity using automated optokinetic reflex tracking in an OptoDrum device with the corresponding software (Striatech). Briefly, mice were placed on an elevated platform surrounded by monitors and a stripe pattern with maximum contrast and constant rotation speed (12°/s) was presented. The optokinetic response was automatically detected and analysed by the software in an unbiased manner and the stimulus pattern (cycles) was continuously adjusted to find the threshold of the animal’s visual acuity.

### Accelerating Rotarod analysis

Mice were placed on a Rotarod Advanced system (TSE systems), and the time on the constantly accelerating rod (5–50 rpm; max latency 300 s) was measured in five consecutive runs per trial as described previously.^7^ Mice were trained with two trials on two consecutive days and measured in a third trial on the third day.

### Spectral domain optical coherence tomography (OCT)

Mice were subjected to OCT imaging with a commercially available device (Spectralis OCT; Heidelberg Engineering) and additional lenses as described previously.^7^^,^^18^ Mice were measured at different ages for longitudinal analysis and the thickness of the innermost retinal composite layer comprising nerve fibre layer (NFL), ganglion cell layer (GCL) and inner plexiform layer (IPL) were measured in high-resolution peripapillary circle scans (at least 10 measurements per scan) by an investigator unaware of the genotype and potential treatment condition of the mice.

### Histochemistry and immunofluorescence

Immunohistochemistry was performed on a 10-µm-thick longitudinal optic nerve cryo-sections after post-fixation in 4% PFA in PBS or ice-cold acetone for 10 min. Sections were blocked using 5% bovine serum albumin (BSA) in PBS and incubated overnight at 4°C with one or an appropriate combination of up to two of the following antibodies: rat anti-CD4 (1:1000, Bio-Rad AbD Serotec MCA1767), rat anti-CD8 (1:500, Bio-Rad AbD Serotec MCA609G), rat anti-CD11b (1:100, Bio-Rad AbD Serotec MCA74G), rat anti-CD169 (binding to sialoadhesin, Sn; 1:300, Bio-Rad AbD Serotec MCA947G), mouse anti-SMI32 (1:1000, BioLegend 801701) and mouse anti-GFAP (1:1000, Sigma-Aldrich G-3893). Immune reactions were visualized using fluorescently labelled (1:300, Dianova) secondary antibodies or biotinylated secondary antibodies (1:100, Vector Laboratories) and streptavidin–biotin–peroxidase (Vector Laboratories) complex using diaminobenzidine–HCl and H_2_O_2_ and nuclei were stained with 4′,6-diamidino-2-phenylindole (Sigma-Aldrich). Light and fluorescence microscopic images were acquired using an Axio imager.M2 microscope (Zeiss) with ApoTome.2 structured illumination equipment, attached Axiocam cameras and corresponding software (ZEN 2.3 blue edition). Images were minimally processed (rotation, cropping and addition of symbols) for the generation of figures using Photoshop CS6 (Adobe). For quantification, immunoreactive profiles were counted in at least three nonadjacent sections for each animal and related to the area of these sections using the cell counter plugin in Fiji/ImageJ (National Institutes of Health).

For quantification of retinal ganglion cells, perfusion fixed eyes were enucleated and specific markers of the inner retinal cell types were labelled in free-floating retina preparations. Fixed retinae were frozen in PBS containing 2% Triton X-100, thawed, washed and blocked for 1 h using 5% BSA and 5% donkey serum in PBS containing 2% Triton X-100. Retinae were incubated overnight on a rocker at 4°C with the following antibodies: guinea pig anti-RBPMS (1:300, Merck ABN1376) and goat anti-Brn3a (1:100, Santa Cruz sc-31984). Immune reactions were visualized using fluorescently labelled (1:500, Dianova) secondary antibodies, retinae were flat-mounted and the total retinal area was measured.

Immunoreactivity against GFAP+ astrocytes in the optic nerves was assessed using a semi-automated thresholding image analysis, according to previously published protocols.^6^^,^^19^

### Experimental design and statistical analysis

All quantifications and clinical analyses were performed by investigators unaware of the genotypes and potential treatment condition of the respective mice after concealment of these data with individual uniquely coded labels. Animals were randomly placed into experimental or control groups according to genotyping results using a random generator (http://www.randomizer.org; last accessed 2021/03/29). For biometrical sample size estimation, the program G*Power (version 3.1.3) was used.^20^ Calculation of appropriate sample size groups was performed in *a priori* power analysis by comparing the mean of two groups with a defined adequate power of 0.8 (1–beta-error) and an α-error of 0.05. To determine the prespecified effect size *d*, previously published data were considered as comparable reference values.^6^^,^^9^ Statistical analysis was performed using PASW Statistics 18 (SPSS, IBM) or GraphPad Prism (version 7) software. Shapiro-Wilk test was used to check for normal distribution of data. The values of *P* considered as significant were indicated by asterisks according to the following scheme: *P < 0.05; **P < 0.01; ***P < 0.001. Significant differences of a respective genotype group in comparison with wild-type mice are indicated above the corresponding group.

### Data availability

All data generated during this study are included in this published article (including Supplementary figure). Further details regarding the presented datasets are available from the corresponding authors upon request.

## Results

### Preventive fingolimod and teriflunomide treatment improves complex clinical phenotypes in CLN1 mice

**Figure 1:**
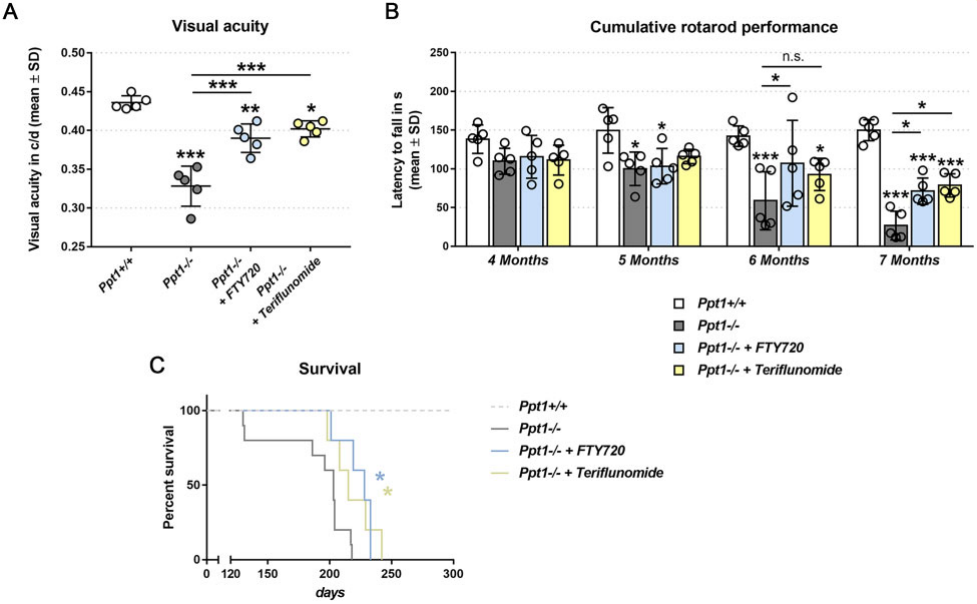
**Preventive fingolimod and teriflunomide treatment improves complex clinical phenotypes in CLN1 mice.** (**A**) Automated optokinetic response analysis of visual acuity (c/d, cycles per degree) of 6-month-old *Ppt1*^+/+^, *Ppt1*^−^^/^^−^, fingolimod-treated *Ppt1*^−^^/^^−^ (+ FTY720) and teriflunomide-treated *Ppt1*^−^^/^^−^ (+ Teriflunomide) mice (circle = mean value of one mouse; *n* = 5 mice per group, one-way ANOVA and Tukey’s *post hoc* test). (**B**) Longitudinal analysis of cumulative Rotarod performance in *Ppt1*^+/+^, *Ppt1*^−^^/^^−^, fingolimod-treated *Ppt1*^−^^/^^−^ and teriflunomide-treated *Ppt1*^−^^/^^−^ mice (circle = mean value of five consecutive runs of one mouse; *n* = 5 mice per group, two-way ANOVA and Tukey’s *post hoc* test). (**C**) Kaplan-Meier survival analysis of *Ppt1*^+/+^, *Ppt1*^−^^/^^−^, fingolimod-treated *Ppt1*^−^^/^^−^ and teriflunomide-treated *Ppt1*^−^^/^^−^ mice (*n* = 5 to 10 mice per group, Log-rank test). *P < 0.05, **P < 0.01, ***P < 0.001.

In the visual system of CLN1 mice, preventive treatment with fingolimod and teriflunomide starting at 1 month of age led to a substantial improvement of histopathological changes.^9^ Here, we tested under the same treatment regime the corresponding clinical readout measure by scoring visual acuity, using automated optokinetic reflex tracking in an OptoDrum device. This readout most likely reflects visual deterioration instead of motor impairment, as the measured head movements *per se* are identical to those of wild-type mice and are strictly dependent on visual input characteristics, like stripe pattern modification. As previously reported,^6^^,^^21^ we found that visual acuity declined significantly in 6-month-old CLN1 mice compared with wild-type mice (Fig. 1A). Importantly, the reduction in visual acuity was attenuated by preventive fingolimod and teriflunomide treatment (Fig. 1A).

Since declining motor skills are another typical clinical feature of CLN1,^3^^,^^22^ we also analysed motor coordination as a disease outcome.

Accelerating Rotarod analysis is an established and reliable measure for motor coordination-related clinical performance.^8^^,^^23^^,^^24^ Longitudinal, monthly analysis of Rotarod performance of untreated CLN1 mice at 4–7 months of age revealed a progressive decline of latencies to fall from the constantly accelerating rod, reflecting impaired motor coordination (Fig. 1B). Preventive treatment with either fingolimod or teriflunomide delayed the decline of motor coordination in the CLN1 mice (Fig. 1B).

Based on our previous observations regarding genetic immune deficiency,^6^^,^^7^ we also tested the possibility that immunomodulatory treatment may increase the longevity of CLN1 mice. Corresponding Kaplan-Meier survival curves showed that the first untreated CLN1 mice died around postnatal day 130, while all CLN1 mice treated with either drug survived until around postnatal day 200 (Fig. 1C). In summary, both immunomodulators significantly, yet mildly, extended the survival of CLN1 mice.

### Therapeutic fingolimod and teriflunomide treatment attenuates neuroinflammation and improves histopathological and behavioural readout measures in CLN1 mice

Since diagnosis for CLN diseases usually occurs when symptoms have already emerged, we next investigated whether treatment with fingolimod and teriflunomide is also of benefit when treatment starts therapeutically at 5 months instead of 1 month of age, followed by analysis at 7 months of age. We focused our analysis on the retinotectal system as an established surrogate system for CNS pathology in CLN diseases.^6^^,^^7^^,^^9^^,^^18^

**Figure 2:**
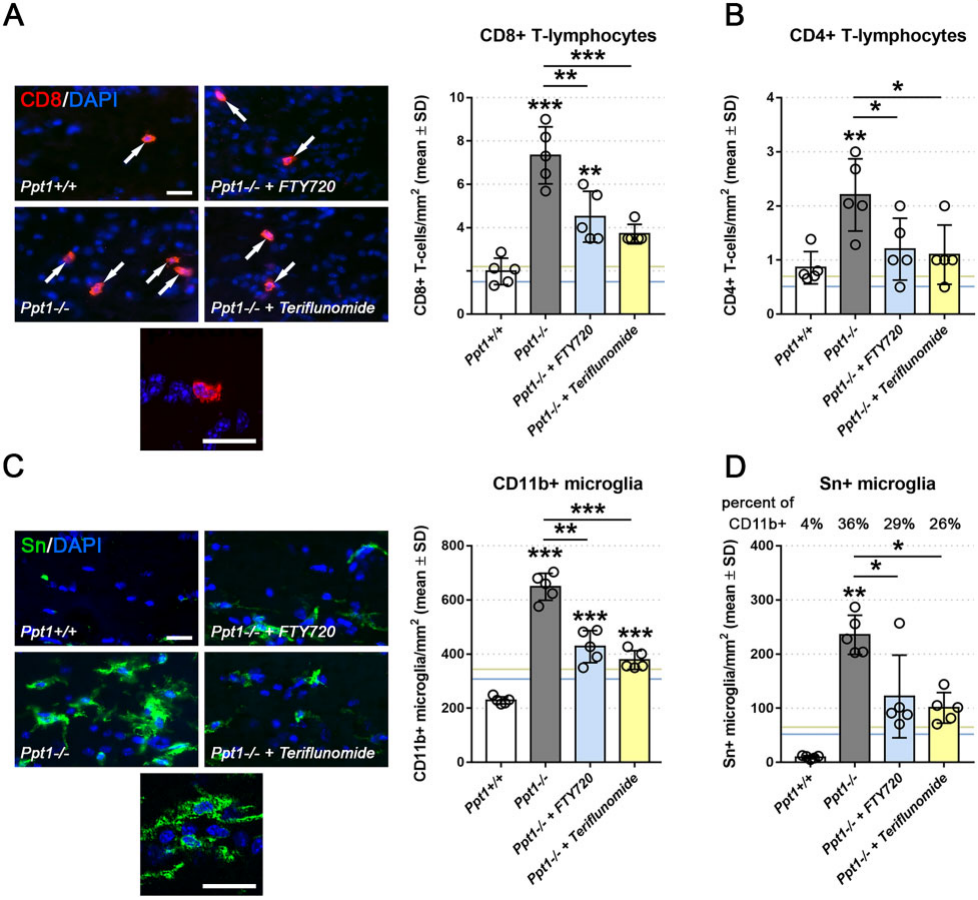
**Therapeutic fingolimod and teriflunomide treatment attenuate neuroinflammation in CLN1 mice.** (**A**) Representative immune fluorescence microscopy and quantification of CD8+ and (**B**) CD4+ T-lymphocytes in longitudinal optic nerve sections from 7-month-old *Ppt1*^+/+^, *Ppt1*^−^^/^^−^, fingolimod-treated *Ppt1*^−^^/^^−^ (+ FTY720) and teriflunomide-treated *Ppt1*^−^^/^^−^ (+ Teriflunomide) mice (circle = mean value of one mouse; *n* = 5 mice per group, one-way ANOVA and Tukey’s *post hoc* test). Scale bars: 20 µm. (**C**) Representative immune fluorescence microscopy and quantification of CD11b+ and (**D**) Sn+ microglia in longitudinal optic nerve sections (*n* = 5 mice per group, one-way ANOVA and Tukey’s *post hoc* test). Blue and yellow lines indicate mean values from previously published preventive (from 1 to 6 months of age) fingolimod and teriflunomide treatment approaches, respectively ^9^. Scale bars: 20 µm. *P < 0.05, **P < 0.01, ***P < 0.001.

At first, we investigated the impact of pharmacological immunomodulation on CNS-resident T-lymphocytes. Both immunomodulators attenuated neuroinflammation by reduction in the recruitment of pathogenic CD8+^6^ and CD4+ T-cells in the therapeutically treated CLN1 model (Fig. 2A and B). We also quantified the number of CD11b+ microglial cells in optic nerves and found a significantly lower number when CLN1 mice were therapeutically treated with the immunomodulators compared with untreated controls (Fig. 2D). Also, pro-inflammatory activation of microglial cells was reduced by both immunomodulators, as reflected by reduced expression of sialoadhesin (Sn) by microglial cells (Fig. 2C and D). However, compared with our previously performed treatment approaches, therapeutic treatment was less efficient than preventive treatment in modulating CNS neuroinflammation (Fig. 2, blue and yellow horizontal lines).^9^ As an additional marker of neuroinflammation, we investigated astrogliosis, a typical feature of CLN diseases and their models,^19^^,^^23^ by GFAP-immunocytochemistry (Supplementary Fig. 1a). Based on this readout parameter, we found reduced astrogliosis upon treatment with both immunomodulators.

**Figure 3:**
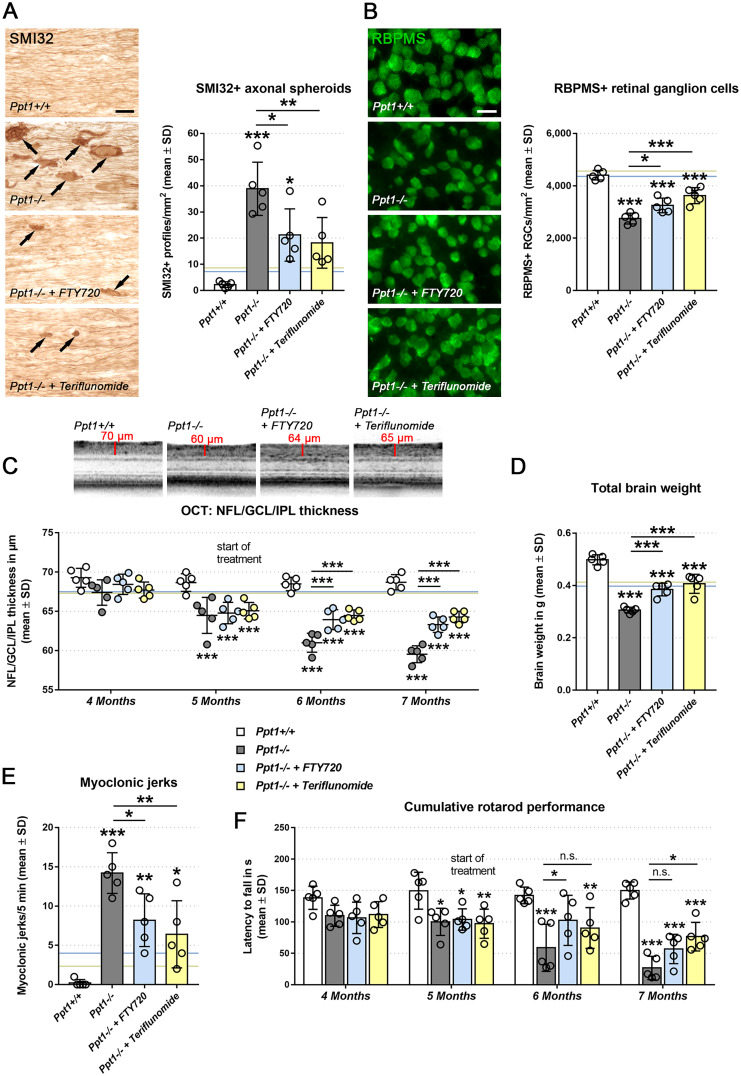
**Therapeutic fingolimod and teriflunomide treatment improves histopathological and behavioural readout measures in CLN1 mice.** (**A**) Representative light microscopy and quantification of SMI32+ axonal spheroids in optic nerves and (**B**) immune fluorescence microscopy and quantification of RBPMS+ retinal ganglion cells in retinae from 7-month-old *Ppt1*^+/+^, *Ppt1*^−^^/^^−^, fingolimod-treated *Ppt1*^−^^/^^−^ (+ FTY720) and teriflunomide-treated *Ppt1*^−^^/^^−^ (+ Teriflunomide) mice (circle = mean value of one mouse; *n* = 5 mice per group, one-way ANOVA and Tukey’s *post hoc* test). Scale bars: 20 µm. (**C**) Representative peripapillary OCT circle scans at 7 months and longitudinal analysis of NFL/GCL/IPL thickness (*n* = 5 mice per group, one-way ANOVA and Tukey’s *post hoc* test). (**D**) Total brain weights of 7-month-old *Ppt1*^+/+^, *Ppt1*^−^^/^^−^, fingolimod-treated *Ppt1*^−^^/^^−^ and teriflunomide-treated *Ppt1*^−^^/^^−^ mice (*n* = 5 mice per group, one-way ANOVA and Tukey’s *post hoc* test). (**E**) Quantification of myoclonic jerks in 7-month-old *Ppt1*^+/+^, *Ppt1*^−^^/^^−^, fingolimod-treated *Ppt1*^−^^/^^−^ and teriflunomide-treated *Ppt1*^−^^/^^−^ mice (circle = mean value of one mouse; *n* = 5 mice per group, one-way ANOVA and Tukey’s *post hoc* test). (**F**) Longitudinal analysis of cumulative Rotarod performance (circle = mean value of five consecutive runs of one mouse; *n* = 5 mice per group, two-way ANOVA and Tukey’s *post hoc* test). Blue and yellow lines (in b–e) indicate mean values from previously published preventive (from 1 to 6 months of age) fingolimod and teriflunomide treatment approaches, respectively. ^9^ *P < 0.05, **P < 0.01, ***P < 0.001.

As a histopathological readout parameter reflecting axonal perturbation, we quantified SMI32+ axonal spheroids in longitudinal sections of optic nerves (Fig. 3A and B). While in untreated CLN1 mice axonal spheroids were frequently detectable (Fig. 3A and B), fingolimod and teriflunomide significantly reduced their numbers (Fig. 3B), albeit less efficiently when compared with preventively treated CLN1 mice.^9^

Next, we focused on the effect of pharmacological immunomodulation on neuron survival and quantified RBPMS+ retinal ganglion cells in flat-mount preparations (Fig. 3A and B). Also in this compartment of the visual system, neurodegeneration was robust in untreated CLN1 mice as reported previously,^6^^,^^7^^,^^9^ while it was mitigated by therapeutic treatment with fingolimod and teriflunomide (Fig. 3B). However, neither of the drugs reached the same level of improvement as in preventively treated CLN1 mice.^9^

We also focused on retinopathy applying optical coherence tomography (OCT) for live imaging of retinal layers as described.^9^^,^^18^ Corroborating our previous findings, *Ppt1*^−^^*/*^^−^ mice showed a progressive thinning of the inner retinal composite layers from 5 months of age onwards (Fig. 3C). Retinal thinning was halted by therapeutic treatment with fingolimod and teriflunomide.

We also analysed total brain atrophy to identify generalized effects of immunomodulatory therapy, which revealed profoundly reduced brain weights of CLN1 mice. This robust decline of brain weight was significantly reduced by both drugs, with similar efficacy as in the previous preventive treatment regime (Fig. 3D). Moreover, cerebellar atrophy showed a trend towards mitigation upon immunomodulatory treatment (Supplementary Fig. 1b). Of note, as in our previous studies,^6^^,^^9^ the accumulation of autofluorescent storage material appeared not to be affected by dampening inflammation (not shown), indicating that neuroinflammation is a secondary phenomenon downstream of the primary lysosomal perturbation.

Next, we investigated whether therapeutic treatment also improved clinical readout parameters. Typical CLN1-related clinical features are myoclonic jerks, both in patients and in animal models. Therapeutic treatment with either drug reduced the frequency of myoclonic jerks significantly (Fig. 3E). Finally, we investigated whether motor coordination is influenced by therapeutic treatment using accelerating Rotarod analysis as a readout. Therapeutic treatment with either fingolimod or teriflunomide delayed the motor decline in the CLN1 mice (Fig. 3F). Although the ameliorating effect was significant at distinct ages, therapy with either drug did not reach the same level of improvement as in preventively treated CLN1 mice (compare Fig. 3F with Fig. 1B).

**Figure 4:**
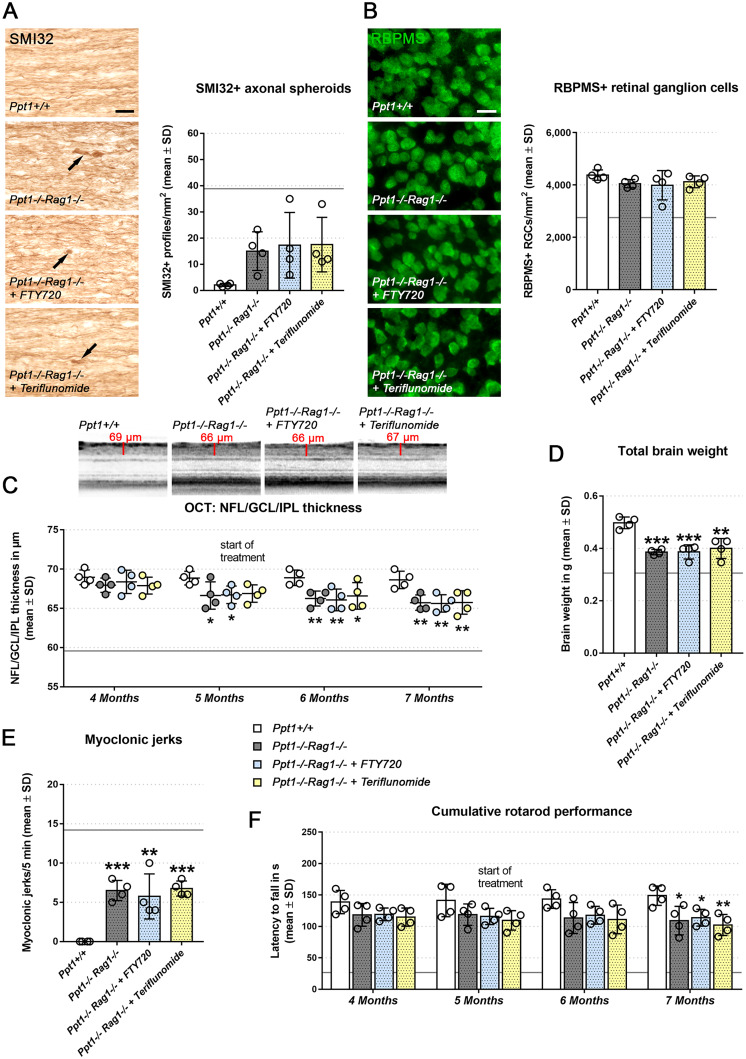
**Treatment effects of fingolimod and teriflunomide are predominantly immunomodulatory.** (**A**) Representative light microscopy and quantification of SMI32+ axonal spheroids in optic nerves and (**B**), immune fluorescence microscopy and quantification of RBPMS+ retinal ganglion cells in retinae from 7-month-old *Ppt1*^+/+^, *Ppt1*^−^^/^^−^*Rag1*^−^^/^^−^, fingolimod-treated *Ppt1*^−^^/^^−^*Rag1*^−^^/^^−^ (+ FTY720) and teriflunomide-treated *Ppt1*^−^^/^^−^*Rag1*^−^^/^^−^ (+ Teriflunomide) mice (circle = mean value of one mouse; *n* = 4 mice per group, one-way ANOVA and Tukey’s *post hoc* test). Scale bars: 20 µm. (**C**) Representative peripapillary OCT circle scans at 7 months and longitudinal analysis of NFL/GCL/IPL thickness (*n* = 4 mice per group, one-way ANOVA and Tukey’s *post hoc* test). (**D**) Total brain weights of 7-month-old *Ppt1*^+/+^, *Ppt1*^−^^/^^−^*Rag1*^−^^/^^−^, fingolimod-treated *Ppt1*^−^^/^^−^*Rag1*^−^^/^^−^ and teriflunomide-treated *Ppt1*^−^^/^^−^*Rag1*^−^^/^^−^ mice (*n* = 4 mice per group, one-way ANOVA and Tukey’s *post hoc* test). (**E**) Quantification of myoclonic jerks in 7-month-old *Ppt1*^+/+^, *Ppt1*^−^^/^^−^*Rag1*^−^^/^^−^, fingolimod-treated *Ppt1*^−^^/^^−^*Rag1*^−^^/^^−^ and teriflunomide-treated *Ppt1*^−^^/^^−^*Rag1*^−^^/^^−^ mice (circle = mean value of one mouse; *n* = 4 mice per group, one-way ANOVA and Tukey’s *post hoc* test). (**F**) Longitudinal analysis of cumulative Rotarod performance (circle = mean value of five consecutive runs of one mouse *n* = 4 mice per group, two-way ANOVA and Tukey’s *post hoc* test). Note that none of the investigated parameters was further improved by either of the immunomodulators in comparison to untreated *Ppt1*^−^^/^^−^*Rag1*^−^^/^^−^ mice. Grey horizontal lines in all graphs indicate mean values of immune competent *Ppt1*^−^^/^^−^*Rag1*^+/+^ mice. *P < 0.05, **P < 0.01, ***P < 0.001.

Finally, we considered that fingolimod- and teriflunomide-based amelioration of histopathological changes and clinical features may not exclusively be caused by the immunomodulatory effects of the drugs. Multiple non-immune effects on neural cells of the drugs were reported previously.^25–29^ We, therefore, tested the possibility of non-immune effects of the drugs by therapeutically treating *Rag1*-deficient CLN1 mice lacking mature T- and B-lymphocytes (Fig. 4). As expected from previous studies in *Rag1*-deficient CLN1 mice,^6^ untreated *Rag1-*deficient CLN1 mice showed improved histopathological features (Fig. 4A–C), less reduced brain weight (Fig. 4D) and ameliorated clinical outcome measures (Fig. 4E and F) in comparison with CLN1 mice with the intact immune system. Importantly, these remaining (mild) histopathological features in immunodeficient CLN1 mice could not be further ameliorated by either fingolimod or teriflunomide, reflecting a predominant immunomodulatory, beneficial effect of the drugs in CLN1 mice.

## Discussion

In a previous study, we have shown that both fingolimod and teriflunomide treatment mitigates retinal thinning, axon degeneration, neuron loss and brain atrophy in CLN1 and CLN3 models.^9^ Additionally, in the CLN1 model, both drugs significantly reduced clinical features strongly related to this disease subform, like myoclonic jerks. Here, in the same model, we analysed treatment effects regarding more complex clinical features, like visual acuity. While visually evoked potentials might have been one possible readout measure for visual performance,^30^ we here selected automated optokinetic response analysis in an OptoDrum device due to the comparability with a previous study from our laboratory.^6^ We found a robust mitigating effect on the impairment of visual acuity by pharmacological immunomodulation, corroborating previous studies investigating the consequences of genetically mediated inactivation of the adaptive immune system.^6^ Based on our previous histopathological studies, this improved clinical outcome may be caused by a rescue of retinal ganglion cell axons and the corresponding cell somata.^6^^,^^9^ Additionally, it is conceivable that a potential rescue of other retinal neurons degenerating in CLN1 models, like photoreceptors and bipolar cells,^31^ might contribute to improved visual acuity upon immunomodulation. However, the impact of the adaptive immune system on these neuronal populations remains to be investigated.

Another readout measure for complex clinical disease progression is analysis, as it faithfully reflects motor coordination, which is typically impaired in CLN1 patients.^3^^,^^22^ Initially used in the CLN1 model to longitudinally investigate deterioration of motor performance and correlating with cerebellar atrophy,^8^^,^^23^ we here apply this technique to measure the clinical outcome effects of fingolimod and teriflunomide treatment longitudinally. We found indeed a robust delay in decline of motor performance by treatment with both drugs, correlating with our previously identified reduction in brain^9^ and cerebellar atrophy upon immunomodulatory treatment. It is plausible to assume that among the preserved neurons are cerebellar Purkinje cells and neurons of the motor cortex, somatosensory cortex, thalamic nuclei and basal ganglia.^19^^,^^23^

Since the genetic attenuation of neuroinflammation by the innate^7^ or adaptive immune system^6^ extended life span in CLN1 mice, we also investigated the impact of pharmacological immunomodulation on this outcome. Pharmacological reduction of cytokine production in glial cells by the small molecule MW151 did not lead to the extension of life span in CLN1 mice.^8^ Here, we present a mild, yet statistically significant increase in longevity of CLN1 mice treated with fingolimod or teriflunomide.

Despite significant effect on life span by immunomodulatory therapy, care should be taken when interpreting or even translating this finding to human patients. Of note, *Ppt1*-deficiency in mice normally reduces life span by less than 70%, while in CLN1 patients, life span is reduced by nearly 90%, with an average life expectancy of around 9 years. Due to these basic differences in relative longevity between the murine CLN1 model and CLN1 patients, it is presently difficult to estimate life-extending effects by immunomodulation. Moreover, premature death of CLN1 mice might also be influenced by other non-neurological and neuroinflammation-independent deficits. Thus, we would rather view these pharmacological approaches as an attempt to improve life quality (e.g. by rescuing visual acuity) and to reduce suffering than to raise too much hope for a substantially increased life span. To get an idea about the efficacy of treatment approaches on longevity in general, models of larger size, like spontaneous canine CLN1 models (see Refs.^32–34^ for review) or a recently generated ovine CLN1 model with a similarly reduced longevity as human patients^35^ may be more suitable than mice.

Another important goal of our study was to investigate whether therapeutic pharmacological immunomodulation with fingolimod and teriflunomide mitigates disease even when neurodegeneration is already ongoing and clinical symptoms are detectable. This therapeutic approach is of high clinical relevance, as CLN diseases are usually diagnosed after disease onset.

At first, we investigated the impact of treatment on T-lymphocytes, the primary target cells of both immunomodulators. Upon treatment from 5 to 7 months of age, numbers of both CD8+ and CD4+ T-lymphocytes were significantly less elevated in the CNS than in non-treated CLN1 mice. Also, microgliosis was attenuated, most probably by reduced interaction with lymphocytes.^6^^,^^9^ Of note, our present and previous findings show that in untreated CLN1 mice, numbers of CD8+ T-cells are already elevated at 3 months of age and progressively increase in number until the end stages of disease.^6^^,^^9^ Here, we show that, upon therapeutic treatment, this progressive increase in number is halted at levels similar to treatment start at 5 months of age, which correlates with significant attenuation in neurodegenerative changes and clinical outcomes (Fig. 3). For example, upon therapeutic treatment, axonal spheroid formation, neuron loss, retinal thinning and impairment appeared halted near the state of treatment initiation.

In comparison with preventive treatment from postnatal months 1 to 6 of age,^9^ therapeutic treatment appeared less effective regarding T-lymphocyte reduction, dampening of microglia activation and improvement of most neurodegenerative and clinical features (see Figs 2 and 3; blue and yellow horizontal lines). This can only be partly explained by an earlier analysis of preventively treated mice in our previous study. Interestingly, the impact of therapeutic treatment on brain atrophy was comparable with that of preventive treatment (Fig. 3D), possibly due to a later start of damage in some brain regions.^19^ For other CNS regions, such as the retinotectal system, degeneration is already ongoing at the time point when therapeutic treatment has started. Moreover, immunomodulation may not be as effective under ongoing neuroinflammatory conditions when increased numbers of adaptive immune cells have already been recruited to the CNS. This may be particularly related to the mode of action of fingolimod, impairing emigration of lymphocytes from secondary lymphoid organs and the subsequent population of the CNS.^10^ Indeed, clinical studies showed that fingolimod is unable to slow down disease progression in progressive forms of MS,^36^ as opposed to the cytostatic teriflunomide stabilizing or even improving chronic, long-term disability in different MS forms.^37^^,^^38^ Nevertheless, both drugs succeed in halting the progressive increase in neuroinflammation when applied at a symptomatic stage of disease, leading to improved neurodegenerative and clinical outcome. Whether in the CLN1 model teriflunomide also favours increase in disease dampening CD8+/CD122+ regulatory T-lymphocytes as in other CNS mutants^39^ remains to be shown.

Although both drugs used in this study considerably differ regarding their immunomodulatory mechanisms, they had remarkably similar outcomes in preventive and therapeutic treatment. For both drugs pleiotropic, non-immunological functions have been postulated.^25–29^ Here, we demonstrate that the observed beneficial effects of the drugs in CLN models are most likely immunomodulatory, since both fail to further improve the remaining mild pathology in *Rag1-*deficient CLN1 mice,^6^ but fully recapitulated beneficial effects of *Rag1*-deficiency on histopathological and clinical outcome. This finding is of substantial clinical impact since it opens the possibility to consider newly emerging immunomodulators of even higher specificity or efficacy than the here applied ones for therapy in CLN diseases. Presently, however, it might be a feasible strategy to initiate clinical trials with young CLN1 patients that are already symptomatic using either of the established drugs. The fact that fingolimod is well tolerated in pediatric MS patients^40^ and hopefully also other established immunomodulators presently under respective trials, including teriflunomide,^41^ gives hope that immunomodulation could become a potent option to make the presently untreatable, rapidly progressing CLN diseases more bearable for patients and their relatives.

## Supplementary material

Supplementary material is available at *Brain Communications* online.

## Supplementary Material

fcab047_Supplementary_Data

## Abbreviations

B-lymphocyte =: bursa-(equivalent organ-) derived lymphocyte
CD =: cluster of differentiation
CLN =: ceroid lipofuscinosis, neuronal
FTY720 =: synonym for Fingolimod, with no direct translation
GCL =: ganglion cell layer
GFAP =: glial fibrillary acidic protein
IPL =: inner plexiform layer
MW151 =: small molecule therapeutic targeting inflammation, with no direct translation
NCL =: neuronal ceroid lipofuscinosis
NFL =: nerve fibre layer
OCT =: optical coherence tomography
PBS =: phosphate-buffered saline
PFA =: paraformaldehyde
PPT1, *Ppt1* =: palmitoyl-protein thioesterase 1
*Rag1* =: recombination activating gene 1
RBPMS =: RNA-binding protein with multiple splicing
S1P =: sphingosine-1-phosphate
SMI32 =: monoclonal antibody label ling non-phosphorylated neurofilament H, with no direct translation
Sn =: sialoadhesin
T-lymphocyte =: thymus-derived lymphocyte
