# Supplementary material for: Immune modulation attenuates infantile neuronal ceroid lipofuscinosis in mice before and after disease onset
Source: Brain Commun. 2021 Mar 21;3(2):fcab047. doi: 10.1093/braincomms/fcab047 (PMC8098642; doi:10.1093/braincomms/fcab047)
Supplement: fcab047_Supplementary_Data [file fcab047_supplementary_data.pdf]

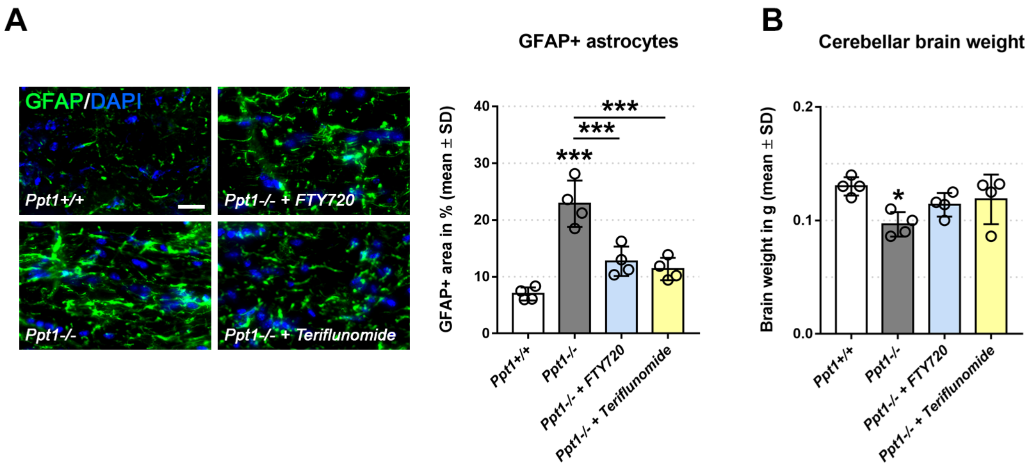

Supplementary Figure 1 Therapeutic treatment with fingolimod and teriflunomide reduces astrogliosis and leads to a trend towards mitigated cerebellar atrophy. (**A**) Representative immune fluorescence microscopy and quantification of GFAP+ astrocytes in optic nerves and (**B**) cerebellar brain weights of 7-month-old *Ppt1+/+*, *Ppt1-/-*, fingolimod-treated *Ppt1-/-* (+ FTY720) and teriflunomide-treated *Ppt1-/-* (+ Teriflunomide) mice (n = 4 mice per group, One-way ANOVA and Tukey's *post hoc* test). *$P < 0.05$, ***$P < 0.001$.
